# Reimagining publics and (non) participation: Exploring exclusion from science communication through the experiences of low-income, minority ethnic groups

**DOI:** 10.1177/0963662517750072

**Published:** 2018-01-10

**Authors:** Emily Dawson

**Affiliations:** University College London, UK

**Keywords:** exclusion, public participation, science communication, science museums, social justice, television

## Abstract

This article explores science communication from the perspective of those most at risk of exclusion, drawing on ethnographic fieldwork. I conducted five focus groups and 32 interviews with participants from low-income, minority ethnic backgrounds. Using theories of social reproduction and social justice, I argue that participation in science communication is marked by structural inequalities (particularly ethnicity and class) in two ways. First, participants’ involvement in science communication practices was narrow (limited to science media consumption). Second, their experiences of exclusion centred on cultural imperialism (misrepresentation and ‘Othering’) and powerlessness (being unable to participate or change the terms of their participation). I argue that social reproduction in science communication constructs a narrow public that reflects the shape, values and practices of dominant groups, at the expense of the marginalised. The article contributes to how we might reimagine science communication’s publics by taking inclusion/exclusion and the effects of structural inequalities into account.

## 1. Introduction

Abdou: You know when you start thinking of going [out], the science museum or a museum is the last place that you would even think of, that you would even consider on your list, even if you were doing a list of hundreds of places that you want to go.

Emily: It wouldn’t even be on the list?

Abdou: Not on the list!

Emily: So why is that?

Abdou: You cannot connect with it. It’s for those people that it matters to […] the museum, or science itself. (Sierra Leonean Group)

Questions about publics for science communication, their practices and their attitudes, have been much discussed, not least in this journal. While in the last 30 years the public have become plural and heterogeneous ‘publics’ in the science and society literatures, this nod towards plurality does not tell the full story. What does it mean to be excluded from science communication practices that, as Abdou, argued in the interview extract above, are only ‘for those people that it matters to’?

A driving assumption in research on science and society relationships is that science communication is a good thing. While participation in science communication can have both societal and individual benefits, these benefits are contested. Thus, at the societal level, science communication can be seen as part of deliberative, participatory democracy that improves policy decisions, while also criticised as simply a capitalist feature of selling science (Stilgoe et al., 2014; Thorpe and Gregory, 2010). Non-participation is problematic for societies in a normative sense, therefore, since it may impair political and market processes. At the personal level, participation in science communication is thought to benefit people by sharing valuable knowledge, by opening up science policy practices or as a key element of our culture (Davies and Horst, 2016; Delgado et al., 2011). Questions remain, however, at both scales, about whose values, knowledge and culture such practices reproduce.

The landscape of science communication is a shifting and fractured one, ranging from politically oriented activities (such as policy consultations) to those with cultural or educational motivations (such as museum visits or television programmes). As a result, in this article, I take a broad view of science communication (Davies and Horst, 2016). A broad approach also left space for participants to describe science communication in their own terms rather than using a strict definition. I use theories of social justice and social reproduction to explore how access to and involvement in dominant modes of representation and communication in a society, such as science communication, are forms of power (Bourdieu, 1984; Young, 1990). This collection of concepts is useful because they allow questions of inequality to be brought explicitly into focus. While this approach is used in cultural studies to explore participation in arts and cultural practices, it is rarely used to look at participation in science-related cultural practices (Bennett et al., 2009; Prieur and Savage, 2013).

This article contributes to debates about science and society relationships by exploring experiences of science communication from those on the outskirts of such practices to reimagine such publics. I argue that science communication practices are shaped by structural inequalities and, as a result, are far from public. Although this criticism may seem obvious, I draw on data from 66 people from low-income, minority ethnic backgrounds to map their participation (or non-participation) in science communication and how they perceive their inclusion/exclusion. In particular, I argue that science communication practices construct a narrow public that reflects the shape, values and practices of dominant groups. This article is organised around two research questions. First, in what kinds of science communication practices were participants involved, or more simply, what did they do or not do? Second, how did they experience exclusion from science communication?

## 2. Imagining excluded publics for science communication

Understanding how publics are imagined as excluded from science communication has a troubled (racist/sexist/classed) history in public understanding of science research. While studies of science and society relationships have long debated questions of deficits, even the more nuanced models of public knowledge and attitudes towards science often overlook the roles played by structural inequalities and the intersections of ‘race’/ethnicity, gender, class and other social positions (see, for example, Sturgis and Allum, 2004). For example, although science communication practices have been described as ‘sharply unevenly distributed’ (Rommetveit and Wynne, 2017: 134), questions of social justice, equity and exclusion are rarely discussed. This is important because just as social research methods contribute to ‘making’ publics and influence how their practices are understood, so too has research constructed publics and participation in relation to science and science communication (Michael, 2012; Savage, 2010). Indeed, the concept of an ‘imagined public’ has become part of the public understanding of science lexicon (Marris, 2014; Rommetveit and Wynne, 2017). However, excluded or non-participating publics have remained largely unexamined in research or have been imagined in negative terms.

Where can we find publics who do not participate in science communication activities in the research literature? Despite a wealth of qualitative case studies exploring specific science communication activities and their associated publics, comparatively little research examines what it means not to participate in such practices (see, for example, Dawson, 2014a). In contrast, large-scale surveys of public attitudes to our knowledge of science do identify publics through their non-participation in science communication, albeit in problematic ways (Burns and Medvecky, 2016). Take, for example, the most recent of the UK Public Attitudes to Science (PAS) survey reports (Ipsos MORI, 2014). Two segments of the public identified in the report demonstrate the negative construction of excluded publics. The segments (the ‘concerned’ and ‘disengaged sceptics’ (Ipsos MORI, 2014: 134)) describe people who felt ill informed about science, did not trust science or scientific regulation and rarely participated in science communication activities.^1^ Notably, these segments included comparatively higher proportions of people from minority ethnic backgrounds, socio-economically disadvantaged backgrounds and women. While such surveys follow in the tradition of earlier *Public Understanding of Science* scholarship that sprang from concerns about public disinterest in science, they reveal troubling assumptions about deficits that shape how such publics and their practices are understood.

Applying concepts from social justice to the PAS segments highlights how damning such analyses are. Structural inequalities – injustices that result from both people’s unquestioned biases and oppressive features of political, cultural, educational or market forces – disadvantage certain groups in ways that can overlap or intersect (Crenshaw, 1991; Young, 1990). Indeed, looking another way at the PAS data in combination with other surveys of science centre and museum visitors shows White, middle-class people living with their families in urban areas are more likely to participate in activities ranging from visits to botanic garden, aquaria and museums to science talks or science festivals (Department for Culture Media and Sport, 2011; Ecsite-UK, 2008; Ipsos MORI, 2011, 2014). Data from the United States mirror these patterns; those most likely to participate in science communication activities, such as visits to museums, zoos or aquaria are more educated, earn more money and have young children in their household (Bell et al., 2009; National Science Foundation, 2012). In other words, in these countries, participants for these science communication activities are drawn from socially dominant groups. We can also infer from such data, who is less likely to participate in science communication: people from socio-economically disadvantaged backgrounds and ethnic minorities (Dawson, 2014a; Dawson, 2014b; Dawson 2017; Feinstein and Meshoulam, 2014). These patterns of participation raise questions about how the experiences of the ‘concerned’ and ‘disengaged sceptics’ are imagined.

Without taking structural inequalities into account, ideas about non-participation – whether in culture or politics, science or arts – often imply, as Levitas (2004) has argued, that excluded people ‘have the wrong values and attitudes’ (p. 49). By describing people in terms of perceived deficiencies (lack of appreciation, knowledge and participation), analyses like the PAS segmentation described above condemn excluded publics and their practices while sidestepping issues of structural inequality. Thus, the ‘concerned’ and the ‘disengaged sceptics’ are framed as the problem (the wrong attitudes, values and practices when it comes to science communication), rather than examining whether science communication practices are exclusive. As a result, publics that do not or cannot participate in science communication are imagined through such research as deficient and responsible, at least in part, for their own exclusion.

Combining ideas about social justice and publics for science communication is, however, not straightforward. Inclusion agendas are renowned for reifying dominant practices and values with little regard for the needs, interests or practices of marginalised groups (Levitas, 2004). For instance, dominant cultural practices (whether arts or science based) have long been critiqued as a form of ‘moral regulation’ (McGuigan, 1996: 16). Furthermore, as Solomon (2012) noted, science has been uncritically framed in culture, education and politics as ‘especially good for you’ (p. xiii). Overlapping normative assumptions about dominant culture, politics and science can therefore frame inclusion in science communication as a form of crusade, one that seeks to generate larger publics for science, while remaining fundamentally uncritical of science communication practices or how publics are constructed (Dawson 2014c; Dawson 2017; Lee and Buxton, 2010). How then might we better understand publics and their non-participation in or exclusion from science communication?

## 3. Theoretical background

In this article, I use two sets of theories to understand experiences of exclusion from science communication. First, I work with Bourdieu’s (1984) theory of social reproduction, which I use to explore participants’ involvement in science communication. Second, I draw on theories of social justice to understand participants’ experiences of exclusion, alongside whom and what could be considered public.

Much of the research on social reproduction and cultural participation, including that of Bourdieu, focuses on the arts. I build here on Hesmondhalgh’s (2006) reminder that Bourdieu’s field of cultural production included law and science alongside the arts and extend these ideas to science communication. Bourdieu (1984, Bourdieu and Johnson, 1993) argued that forms of educational and cultural participation are socially stratifying practices. In other words, they maintain social hierarchies by reproducing patterns of advantage and disadvantage through who can and cannot participate. He unpacks this idea using the concepts of capital, field and habitus. Cultural capital – the idea used most here – can be understood as knowledge and familiarity with field-specific practices resulting in competency (Bourdieu and Darbel, 1991; Skeggs, 2004). It can be used, built or lost in specific contexts (fields) depending on how you have been socialised into that field through previous experiences (habitus). For instance, in a science context, studies have shown that what Pandora and Rader (2008: XX) called ‘the scientific imagination’ is a valuable thinking practice, can be considered ‘science capital’ and developed through taking part in science communication practices (Archer et al. 2015; Kato-Nitta, 2013). In other words, scientific knowledge (whether about content, practices or applications) is a valuable form of cultural capital.

Scholars argue that social reproduction happens in at least two ways. For Bourdieu and Johnson (1993), restricted access to production and participation in a given field is a sign of the prestige associated with that field. Thus, scholars have framed cultural practices along spectrums of social privilege as, for instance, high-brow or low-brow, elite versus popular, and traditional versus everyday. Typically, high, elite or traditional practices are more restricted and use dominant forms of cultural capital (McGuigan, 1996). Miles and Gibson (2016) refer to this perspective as the orthodox approach to understanding cultural participation since it reflects classed, gendered and racist assumptions about how certain practices are valued while others are not. Other scholars argue that participation across practices, from the elite to the popular, or being a ‘cultural omnivore’, marks social reproduction (Bennett et al., 2009). From this perspective, being able to participate in and accrue cultural capital across different practices is advantageous. Thus, while everyone has cultural capital, not all forms or practices are equally valued or legitimated depending on the field and the relative status of an individual (Skeggs, 2004).

Theories of social justice are a useful second lens to examine how social reproduction is related to power and oppression. Understanding how disadvantages are reproduced in relation to practices like science communication is not as straightforward as looking for a person or organisation to blame. Rather, structural inequalities (biases, such as sexist assumptions about aptitude for science or policies about which migrants are considered ‘legal’) are embedded across institutions, policies and practices, as well as our everyday behaviours, in ways that maintain or exacerbate social inequalities (Fraser, 2003; Young, 1990). Oppression is, in this sense, structural and systematic, albeit sometimes hard to pinpoint.

While forms of oppression are multiple and overlapping, I draw on Young’s (1990) theorisation of cultural imperialism and powerlessness because these help to understand what happens if you are not able to build cultural capital or even access a particular field (such as science communication) to begin with. Cultural imperialism, for Young (1990), is experienced when socially dominant perspectives and practices suppress or invalidate the views of marginalised groups. For example, in a science communication context, cultural imperialism could be experienced by people whose cultural artefacts are displayed in ethnographic exhibits without their co-operation and in ways that mark their knowledge, practices and selves as Other (Lavine and Karp, 1991). Powerlessness, as developed by Young (1990), combines issues of ‘race’/ethnicity, gender and class to describe the experience of being disrespected and having little or no autonomy over your choices, for instance, in terms of employment or political voice because of your marginalised social status. In science communication, this could happen when people are not listened to in a consultation exercise or when their opinions are not even sought.

Finally, the concepts of ‘public’ and ‘the public’ are contested in various ways but nonetheless provide a helpful background for framing how inclusion/exclusion can be understood at the societal level in terms of who can access that which is ‘public’ (Benhabib, 2002). In this article, I take the position that ‘public’ means, as Young (1990) succinctly put it, ‘what is open and accessible’ (p. 119). In line with feminist critiques of Habermasian constructions of the public sphere – particularly those concerned with gendered, Eurocentric distinctions between the dualistic public versus private and everyday versus special (Ebrey, 2016; Fraser, 1990) – Young’s view of ‘public’ supports a broad view of science communication. Thus, rather than focusing only on special events outside the home, for example, science communication activities that make science public in Young’s sense could range from television watched at home to taking part in a town hall meeting about local pollution.

I also draw on an inclusive, participatory democratic model of ‘the public’ from social justice theorists whose work has understood publics as heterogeneous and active in global, multicultural societies and has sought to value difference (Benhabib, 2002; Young, 2000). From this perspective, an inclusive, empowering model of science communication would be one that involves multiple voices, spaces and publics in equitable ways. For scholars of social justice, being unable to participate in, benefit from or otherwise shape valued public practices constitutes a significant form of marginalisation and oppression (Fraser, 2003; Young, 1990). Thus, if we consider science communication socially or personally valuable, we must consider issues of inclusion/exclusion.

## 4. Methods

### Study context and participants

This mixed-methods qualitative study followed an ethnographic approach and focused on people likely to be excluded from science communication. Fieldwork sites were identified on the basis that participants were from backgrounds under-represented in science communication, that is, socio-economically disadvantaged backgrounds and minority ethnic backgrounds (Dawson 2014a; Department for Culture Media and Sport, 2011; Ipsos MORI, 2014). This study was carried out in the United Kingdom in the central London boroughs of Southwark and Lambeth, areas with large clusters of socio-economically disadvantaged, minority ethnic people. Five community groups were involved in this study (see Table 1). Groups were grass-roots community groups who coalesced around a common sense of shared cultural heritage. The recruitment and fieldwork in this study were designed to be exploratory, not representative. Participants were mixed in terms of age and educational backgrounds. Between one and five participants in every group were educated to degree level (including one science MSc in the Sierra Leonean group). In each group, however, some adults had no formal qualifications and had not been to school. Participants in every group had children, who I met during fieldwork (but were not included in interviews or focus groups).

**Table 1.** Overview of participants.

Group	Participants’ gender		Age range
	Female	Male	
Afro-Caribbean (*n* = 7)	7	–	One in her 30s, one in her 40s, two in their 50s, two in their 60s
Asian (*n* = 13)	11	2	Two in their 30s, one in his 40s, two in their 50s, five in their 60s, three in their 70s
Latin American (*n* = 19)	11	8	One aged 18, five in their 20s, seven in their 30s, five in their 40s, one in his 50s
Sierra Leonean (*n* = 21)	12	9	One aged 18, two in their 20s, eight in their 30s, seven in their 40s, one in his 50s, two in their 70s
Somali (*n* = 6)	4	2	Three in their 20s, one in his 30s, two in their 40s

As argued earlier, categorising people is not a value-free mechanism; rather, it creates and labels specific kinds of publics, for example, as experts, stakeholders or ‘Others’ (Michael, 2012). Researching exclusion risks labelling participants in similarly problematic ways. Notably, participants’ status as immigrants to the United Kingdom complicates neatly describing them in terms of class and ‘race’/ethnicity, though these were the two driving factors behind their recruitment. For instance, many participants sent money to family members in their countries of origin and described their backgrounds as middle class at ‘home’. In the United Kingdom, however, due to a combination of devalued ‘foreign’ qualifications, limited English language fluency and the structure of the labour market, participants were unemployed or employed in precarious, badly paid jobs as temporary nurses, cleaners or security guards during the project. As a result, I describe participants here as from low-income backgrounds, rather than in class terms, since their migration complicated their socio-economic status. The issue of ‘race’/ethnicity was also complex since, as others have shown, participants’ comparative marginalisation (in terms of access to labour markets, education, culture and politics) in the United Kingdom derived from their migration rather than ‘race’/ethnicity (Sassen, 2001; Vertovec, 2004). No participants were born in the United Kingdom, though around half had lived in the United Kingdom for a significant part of their lives (10 years or more). Thus, it is important to understand ‘race’/ethnicity and class as inextricably linked to the context of migration for participants and alongside the other intersecting subjectivities of their lives.

### Data collection and analysis

Fieldwork took place over 2 years, during which I attended community events and visited participants’ homes to learn about how they saw science communication. Alongside ethnographic observations, I interviewed participants and carried out focus groups (see Table 2). Interviews were conducted throughout the fieldwork period. A life history approach was used for participants’ first interviews and where subsequent interviews were possible these followed up on themes or stories related to views, expectations and experiences of science communication from earlier interviews. Focus groups were semi-structured and explored participants’ involvement with science communication as a group, building on themes emerging from the interview analysis. For instance, participants were asked broadly about their encounters with science communication (as they defined it), their reasons for participation (or not) in science communication activities and photographs were used as discussion prompts for activities participants were less familiar with. Focus groups and interviews were audio recorded, transcribed and anonymised during transcription; pseudonyms are used here.

**Table 2.** Participants and related research methods.

Group	Total number of participants per group	Female	Male	Interviews	Focus groups
Afro-Caribbean	7	7	0	2	1 (*n* = 6)
Asian	13	11	2	4	1 (*n* = 5)
Latin American	19	11	8	12	1 (*n* = 12)
Sierra Leonean	21	12	9	10	1 (*n* = 8)
Somali	6	4	2	4	1 (*n* = 4)
Total	66	45	21	32	5

Data were analysed in iterative cycles, beginning with a detailed reading and annotation of transcripts and field-notes, where I used a content analysis approach to find salient issues and themes (Miles and Huberman, 1994). I moved back and forth between data sets to explore what people talked about, including the instances of overlap, contestation and contradiction. A theoretically driven analysis followed where I applied theories of social reproduction and social justice to the data. Presented below are the findings of these analyses, supported by illustrative data excerpts.

## 5. Analysis and discussion

### Mapping (non)participation in science communication

The answer to the first research question about participation in science communication was simple: participants’ involvement was narrow and limited to popular, everyday practices. Participants’ recognition of and participation in practices they saw as science communication were patterned by what Miles and Gibson (2016) call the orthodox or ‘“official” framework of cultural participation and value in the UK’ (p. 151). That is, the more elite or dominant a practice, the more recognisable it was but the less participants were involved, while ‘low-brow’, popular activities, such as watching television, were more accessible but not seen as important. Indeed, for Bourdieu, cultural practices become legitimate and dominant through state support and visibility (even for those unable or unwilling to participate) combined with restricted access (Bourdieu, 1984; Bourdieu and Johnson, 1993). Thus, the more elite a practice, the more visible it was and the more likely that access was limited.

Museums were the most recognised of all the science communication practices that we talked about. This did not mean, however, that museums were visited. For instance, Kadiatu and Fatimata from the Sierra Leonean group argued that science museums were of little interest or relevance to their community:

Kadiatu: Not science museums, no (laughs)

Fatimata: I would go to the cinema, I would never tell anyone I would go to a museum.

Kadiatu: I would say half of the Sierra Leone community they never just sit down and say, ‘lets go to the science museum’.

Participants recognised traditional science communication practices and institutions, such as museums, as a form of ‘high-brow’ culture and, as a result, broadly unappealing and inaccessible for people like them (for reasons discussed in answer to the second research question). This finding is particularly problematic for arguments about inclusion/exclusion since Bourdieu (1984) argued that the more dominant a practice, the more valuable the cultural capital involved in such practices. Visiting science museums, however, was laughable. As Kemetta, a gatekeeper for the Latin American group, put it, ‘no, they’re not going to museums, no lofty expectations I imagine (laughing)’. By alluding to the elite or ‘lofty’ status of museums, Kemetta’s comment highlights how the social distance perceived between participants and museums reinforced inaccessibility.

Notably, while some participants or their children visited science museums on school visits, these were not fondly remembered, nor were these experiences that catalysed further visits. As Fatima from the Somali group argued, ‘I probably wouldn’t go back to museums any time soon because I was taken there by force [with school]’. Importantly, this point contradicts claims elsewhere in the museum studies literature that school visits to museums facilitate the inclusion of broader publics (see, for example, Hooper-Greenhill et al., 2009). Thus, despite living in central London and being aware of London’s more prestigious science communication institutions, neither participants nor their children used these resources (see also Dawson 2014b and Dawson 2016).

Newer, less traditional science communication practices were invisible and, as a result, inaccessible for participants. Participants had not heard of science talks in cafes or pubs, science festivals, citizen science practices, science storytelling or science discussion events. For example, Khalid, from the Somali group, explained he would not know where to start to find such events, would not know where they took place, what they were or why he would attend. His perspective was widely echoed across all five groups. Thus, despite the proliferation of science communication practices through significant funding in the United Kingdom over recent years, such activities remained invisible to participants. This finding questions the extent to which these new, hybrid forms of science communication reach new audiences, as is sometimes claimed (Bultitude, 2014; Kaiser et al., 2013).

Similarly, participants had no experience of activities that could be considered under the political branch of science communication, such as local government consultations on socio-scientific issues or Citizens Juries. That is, such activities were not seen as traditional, visible political processes, such as voting. Indeed, although participants saw specific socio-scientific issues as part of their lives (such as agriculture or climate change), across all five groups participants struggled to imagine how or why they would influence political decisions on such issues. As Kirin from the Asian group put it, despite her worries about the links between pesticides, preservatives and health, she felt unable to influence government decisions: ‘how to approach government […] some government meeting, some bosses, government, I don’t know who they are’. Here, we begin to see how cultural capital, powerlessness and exclusion work together. Without the institutional knowledge of how to influence science-related decision-making or knowledge of the various consultative practices that exist, Kirin and the other participants felt powerless to influence change.

Turning to the forms of science communication participants used highlights again how their practices were marked by orthodox distinctions between popular and elite forms of culture (Bourdieu, 1984; Miles and Gibson, 2016). Science communication practices perceived as popular or ‘low-brow’, particularly television and the Internet, were used by participants. For instance, regularly watched television programmes about science ranged from blue-chip nature documentaries to shows where science formed part of the fabric of the programme but was not the main focus, such as the comedy series The Big Bang Theory or the detective series Crime Scene Investigation (CSI). Like museums, practices such as watching television were highly visible. Unlike museums, however, these ‘popular’ practices were woven into participants’ everyday lives. As Thomas from the Sierra Leonean group put it,

Thomas: Going to a museum is like, when last did I go to a museum? When last did any of my friends go to a museum? […] There’s so much on the internet and TV, and like if someone says something to do with science to you, I can just go on YouTube, and write it in and have a look, or I can watch a show like, what’s that guys name, he did that series ‘Life’?

Emily: Attenborough?

Thomas: Yeah, David Attenborough, like I found that wicked, that Life series, I think a lot of people must have watched that show.

Thus, in line with Bourdieu and Johnson’s (1993) work on restricted access, popular or everyday practices, such as watching television, were seen as more relevant and more accessible by participants than museums.

Watching television appears, however, to be a ubiquitous cultural practice in the United Kingdom, even for people excluded from other forms of culture (Bennett et al., 2009; Taylor, 2016). While television and the Internet were identified as sites where participants encountered science communication practices, few participants sought out science through these media. Rather, everyday cultural practices around television watching and going online sometimes overlapped with science content. This raises questions about context, dominant practices and forms of cultural capital for participants whose involvement in science communication was limited to television and the Internet (Bourdieu, 1984; Skeggs, 2004). In other words, can watching television provide the same advantages as taking part in a broader range of science communication activities?

Mapping participants’ involvement in and recognition of science communication practices shows, therefore, that participation was narrow (limited to science media consumption) and patterned by classed distinctions about elite/popular practices. Participants were not involved in ‘high-brow’ science communication practices and were thus unable to access and accrue certain dominant forms of cultural capital related to science communication. Furthermore, participants’ involvement in science communication practices was narrow. That is, they were not science communication ‘omnivores’ (Bennett et al., 2009). However, that is not to say participants’ practices were deficient; these were culturally and politically active people. Participants across all five groups were involved in what Erel (2010) termed community-based cultural practices. Furthermore, while politically oriented science communication practices were unknown to participants, many were seasoned political activists for their communities. Thus, the analysis presented here highlights the need to move away from arguments about participatory, cultural or political deficits when imagining audiences who do not or cannot participate in science communication (Levitas, 2004; Miles and Gibson, 2016). Indeed, participants’ experiences suggest the field of science communication mirrors existing patterns of exclusion from ‘high-brow’ culture and politics (Bennett et al., 2009; Bourdieu, 1984; Miles and Gibson, 2016). Science communication practices can therefore be seen as a restricted and exclusive field of cultural production (Bourdieu and Johnson, 1993).

### Understanding (non)participation: Structural inequalities and exclusion

Exploring the second research question about how participants experienced exclusion from science communication found ‘race’/ethnicity, and its intersections with gender and class/income, to be the most salient features of participants’ experiences. While the Bourdieusian lens is helpful for mapping patterns of (non)participation, it is less helpful for details such as how structural inequalities were experienced by participants. I augment it here with theories of social justice to frame participants’ descriptions of exclusion. I argue that participants’ exclusion from science communication can be understood in terms of cultural imperialism and powerlessness (Fraser, 2003; Young, 1990). These two features of oppression highlight how structural inequalities reinforced exclusion from science communication across the intersecting subjectivities of participants’ lives and produced a perception of an imagined, included public against which they positioned themselves.

#### Cultural imperialism

Cultural imperialism – when the culture, views and practices of the socially dominant appeare universal at the expense of the marginalised – was particularly salient for participants in terms of ‘race’/ethnicity (Young, 1990). Participants saw science communication practices as Eurocentric and reproducing racist stereotypes. For instance, participants in the Somali and Sierra Leonean groups described how they resented the perception of Africa as burdened by disease and ‘saved’ by the West in stories about medicine. White saviour narratives are not uncommon across culture, policy and education, but they are profoundly misrepresentative, disempowering and racist (Kendall, 2015).

Issues of ‘race’/ethnicity and cultural imperialism also intersected with gender. For example, three participants from the Sierra Leonean and Asian groups – all nurses – protested the celebration of Florence Nightingale against the comparative invisibility of Mary Seacole (who they saw as a positive example of Black womanhood) on television and in museums:

Hawa: Because in reality, a lot of Africans have done a lot of things that are good in the world. But most of the time when people are talking about history, when you think about science in the museums, they are forgotten. Maybe the only good thing they put about Black people is in nursing, Florence Nightingale and Mary Seacole, or the slavery.

Lucille: Even Mary Seacole, it’s a major example, Florence Nightingale, they portray her so much in the world.

Hawa: And Mary Seacole is forgotten […] because the things they will tell you about are slavery.

Issues of gender and ‘race’/ethnicity are intertwined here as Hawa and Lucille from the Sierra Leonean group described what they saw as a whitewashed, disempowering history in terms of slavery, science, nursing and Black women. Evident in these accounts of cultural imperialism, from a Bourdieusian perspective, is not that participants lacked cultural capital, but that their cultural capital – the stories, practices and knowledge they valued – was not reflected in the science communication landscape as they saw it. Indeed, as Hage (1998) has argued, Whiteness can be understood as a valorised form of cultural capital in dominant cultural practices to the disadvantage of minority ethnic populations.

(Mis)representation mattered in participants’ accounts of exclusion from science communication. For example, Connie from the Afro-Caribbean group felt science museums pigeon-holed Black people via narrow, racist portrayals. In the same vein, she argued science communication tailored to her community during Black history month was tokenistic, stating, ‘we’re not invited the rest of the year!’. Similarly, Maria from the Latin American group remarked that even in an exhibition about Colombian butterflies, the rich science-related cultural history of Colombians was erased. As many have argued, the representation of cultures, knowledge and people reflect deep-seated assumptions about power (Erel, 2010; Kendall, 2015; Lavine and Karp, 1991). Thus, what Connie, Maria and the other participants described can be understood as structural inequalities, in particular, as relations of dominance and oppression reproduced through forms of cultural imperialism embedded in science communication practices.

#### Powerlessness

Powerlessness was the other key feature of participants’ views about exclusion from science communication, both in relation to ‘race’/ethnicity and its intersections with class/income. Indeed, for Young (1990), powerlessness is closely tied to class, particularly for groups with limited political or work-based authority, and who are not respected for their opinions or status. For instance, Fatimata from the Sierra Leonean group argued that science communication practices left people from minority ethnic groups powerless and voiceless. As she angrily stated,

We, Black people, normally think if you asked me if I would like to be part of whatever discussion based on science, to talk to the government for them to listen, we’ll always say, well, they’re not going to listen to us obviously, because we’re minority people.

For Fatimata, science communication was pointless, since her exclusion was predetermined and embedded in the structural inequalities that shaped whose voices were heard and whose were not. Similarly, when describing his exclusion, Ibrahim from the Sierra Leonean group blamed racist practices embedded in science communication. Specifically, he stated that communities like his were excluded in ways he could not influence because science communication practitioners and institutions were ‘not catering for ethnic minority people and they’re not spreading the message to the ethnic minority communities’. In other words, people like him were not part of the imagined public for science communication.

Turning to the intersections of class/income and ‘race’/ethnicity shows how powerlessness affected participation. Just as leisure activities are marked by assumptions about disposable time and income, so too were science communication practices (Coleman and Kohn, 2007). Participants did not have ‘free’ time, nor did they have money to spend on science communication activities because precarious employment was a feature of all participants’ lives, entangled in their migrant status in the United Kingdom (Sassen, 2001). As described in the ‘Methods’ section, participants’ employment was often badly paid and ad hoc. Thus, many participants worked around the clock with little autonomy. As Luis Diego from the Latin American group explained, exploitative work conditions and science communication did not go together: ‘For me it’s difficult to do it [science communication], because I’m working […] all the time. It’s very, very difficult’. For participants, the money and time required to take part in science communication was simply not available, rendering such practices inaccessible in ways that were beyond their control. This finding is notable, since it belies the idea that free entry to science communication practices makes them financially accessible. For instance, many museums in London are free to enter, but participants still did not have the ‘free’ time to visit, highlighting that the economics of participation are more complex than entry costs alone.

#### Imagined publics

Participants imagined a public for science communication that echoed the research reviewed earlier in this article: the included public was expected to be predominantly White and shaped by perceptions of ‘free’ time and money associated with middle and upper classes. As Thomas from the Sierra Leonean group put it, science communication, especially in museums, was for ‘upper, middle classes. Not even just African culture, but working classes in general too, are not really involved in the culture of it’. Thus, while gender issues arose, ‘race’/ethnicity and class were the most salient features of the imagined public for science communication participants expected, and in return, key features that distinguished themselves from such publics.

The institutional Whiteness and middle-class nature of science communication highlighted here echoes patterns found in studies of education, museums and culture more widely (Ahmed, 2012; Hage, 1998; Lavine and Karp, 1991; Ware and Back, 2002). While these features of science communication might not come as a surprise, they worked powerfully to exclude participants. Thus, participants were keenly aware that science communication was configured around Whiteness and middle/upper class values both in practice (the stories told on television or in exhibits, whose voices were heard in policy consultations) and users/audiences in ways that excluded them, despite the superficial appearance of accessibility. As Connie from the Afro-Caribbean group explained, ‘everyone thinks the door is open, but it’s not really, and that’s probably because the people in charge are quite comfortable and don’t want criticism or to have to change’.

## 6. Conclusion

In mapping participants’ limited involvement in science communication and exploring their views of exclusion from such practices, I have argued that structural inequalities mark participation in two key ways. First, patterns of participation in science communication described by participants were narrow, limited to science media consumption at home, and followed an orthodox view of cultural and political participation (Miles and Gibson, 2016). That is, the more dominant a given science communication practice, the less participants were involved. In contrast, participants enjoyed everyday, popular forms of science communication and community-based forms of cultural or political participation. This finding suggests that participation in science communication operates in similar ways to Bourdieu’s (1984; Bourdieu and Darbel, 1991; Bourdieu and Johnson, 1993) theory of social reproduction via arts, education and cultural participation; that restricted access preserves cultural capital for dominant groups through excluding the marginalised. This finding also indicates that constructions of the public sphere that rely on dualistic, gendered and Eurocentric assumptions about which forms of participation count (public/private, everyday/special) need to be reimagined (Ebrey, 2016; Fraser, 1990). Indeed, taking into account a broader sense of ‘what counts’ might help to reimagine ‘who counts’ in more inclusive terms.

Second, as the analyses of cultural imperialism and powerlessness demonstrate, inaccessibility was not the only feature of participants’ exclusion from science communication. Their accounts of misrepresentation, racist or otherwise negative representations and their powerlessness to participate or change the terms of their involvement suggest their exclusion was deeply embedded. Indeed, participants’ accounts of exclusion highlighted an imagined public for science communication as marked by ‘race’/ethnicity (Whiteness) and class (possessing disposable income and ‘free’ time). Thus, their experiences demonstrate that both included and excluded publics are constructed for science communication, following lines of social advantage and structural inequalities. Drawing on concepts from social justice theorists, therefore, science communication cannot be considered public following Young’s (1990) definition based on openness and accessibility. Taking part in science communication is not open to everyone.

The scope of this article is limited to the perspectives and experiences of 66 people from five groups in the United Kingdom over 2 years. As a result, findings cannot be generalised to the experiences or attitudes of other people, whether from similar ethnic or socio-economic backgrounds. Thus while people from White, working-class backgrounds or minority ethnic, middle-class backgrounds may well share some of participants’ experiences of science communication, further research is needed to explore such questions. The argument and data presented here contribute to how we might reimagine publics and their practices in relation to science communication, given the uneven playing field some face (Bennett et al., 2009; Fraser, 2003; Young, 1990). For instance, this study suggests we should not construct certain publics as disengaged because of their attitudes towards science communication practices without taking structural inequalities into account, nor should we blame them for their exclusion (Ipsos MORI, 2014). Indeed, I have argued that social reproduction in science communication constructs a narrow public that reflects the shape, values and practices of dominant groups, at the expense of the marginalised.

In practical terms, inclusion is clearly not as simple as getting more people through the door. Inviting people from minority ethnic and/or socio-economically disadvantaged backgrounds into spaces or practices that reflect dominant values of Whiteness and class privilege, without fundamentally reimagining the practices involved, is clearly insufficient. Instead, an inclusive model is likely to involve multiple voices, spaces and publics. For instance, museums that reimagine collections with marginalised groups in ways that surface their assets (rather than deficits) and do justice to their histories, practices and values may be able to disrupt their role in social reproduction by developing more equitable experiences (Dawson 2014a; Dawson 2017; Yalowitz et al., 2013). Similarly, citizen science practices that combine marginalised community-based cultural or political activities with dominant modes of practice may present a useful way to rework science communication (Aguirre, 2014).

## Notes

1. Defined in the report as science talks, out of school/university activities, visits to zoos, aquaria, planetaria, nature reserves, science centres and museums.
